# Pharmacological interventions for acute attacks of vestibular migraine

**DOI:** 10.1002/14651858.CD015322.pub2

**Published:** 2023-04-12

**Authors:** Katie E Webster, Afrose Dor, Kevin Galbraith, Luma Haj Kassem, Natasha A Harrington-Benton, Owen Judd, Diego Kaski, Otto R Maarsingh, Samuel MacKeith, Jaydip Ray, Vincent A Van Vugt, Martin J Burton

**Affiliations:** Cochrane ENT, Nuffield Department of Surgical SciencesUniversity of OxfordOxfordUK; Wadham CollegeUniversity of OxfordOxfordUK; Cochrane ENTNuffield Department of Surgical Sciences, University of OxfordOxfordUK; Aleppo University HospitalAleppoSyrian Arab Republic; Ménière's SocietyDorkingUK; ENT DepartmentUniversity Hospitals of Derby and Burton NHS Foundation TrustDerbyUK; National Hospital for Neurology and NeurosurgeryLondonUK; Amsterdam UMC, Vrije Universiteit Amsterdam, Department of General PracticeAmsterdam Public Health Research InstituteAmsterdamNetherlands; ENT DepartmentOxford University Hospitals NHS Foundation TrustOxfordUK; University of SheffieldSheffieldUK; Nuffield Department of Surgical SciencesUniversity of OxfordOxfordUK

**Keywords:** Adult, Female, Humans, Male, Anti-Inflammatory Agents, Non-Steroidal, Anti-Inflammatory Agents, Non-Steroidal/therapeutic use, Headache, Migraine Disorders, Tryptamines, Vertigo, Vertigo/drug therapy

## Abstract

**Background:**

Vestibular migraine is a form of migraine where one of the main features is recurrent attacks of vertigo. These episodes are often associated with other features of migraine, including headache and sensitivity to light or sound. The unpredictable and severe attacks of vertigo can lead to a considerable reduction in quality of life. The condition is estimated to affect just under 1% of the population, although many people remain undiagnosed. A number of pharmacological interventions have been used, or proposed to be used, at the time of a vestibular migraine attack to help reduce the severity or resolve the symptoms. These are predominantly based on treatments that are in use for headache migraine, with the belief that the underlying pathophysiology of these conditions is similar.

**Objectives:**

To assess the benefits and harms of pharmacological interventions used to relieve acute attacks of vestibular migraine.

**Search methods:**

The Cochrane ENT Information Specialist searched the Cochrane ENT Register; Central Register of Controlled Trials (CENTRAL); Ovid MEDLINE; Ovid Embase; Web of Science; ClinicalTrials.gov; ICTRP and additional sources for published and unpublished trials. The date of the search was 23 September 2022.

**Selection criteria:**

We included randomised controlled trials (RCTs) and quasi‐RCTs in adults with definite or probable vestibular migraine comparing triptans, ergot alkaloids, dopamine antagonists, antihistamines, 5‐HT3 receptor antagonists, gepants (CGRP receptor antagonists), magnesium, paracetamol or non‐steroidal anti‐inflammatory drugs (NSAIDs) with either placebo or no treatment.

**Data collection and analysis:**

We used standard Cochrane methods. Our primary outcomes were: 1) improvement in vertigo (assessed as a dichotomous outcome ‐ improved or not improved), 2) change in vertigo (assessed as a continuous outcome, with a score on a numerical scale) and 3) serious adverse events. Our secondary outcomes were: 4) disease‐specific health‐related quality of life, 5) improvement in headache, 6) improvement in other migrainous symptoms and 7) other adverse effects. We considered outcomes reported at three time points: < 2 hours, 2 to 12 hours, > 12 to 72 hours. We used GRADE to assess the certainty of evidence for each outcome.

**Main results:**

We included two RCTs with a total of 133 participants, both of which compared the use of triptans to placebo for an acute attack of vestibular migraine. One study was a parallel‐group RCT (of 114 participants, 75% female). This compared the use of 10 mg rizatriptan to placebo. The second study was a smaller, cross‐over RCT (of 19 participants, 70% female). This compared the use of 2.5 mg zolmitriptan to placebo.

Triptans may result in little or no difference in the proportion of people whose vertigo improves at up to two hours after taking the medication. However, the evidence was very uncertain (risk ratio 0.84, 95% confidence interval 0.66 to 1.07; 2 studies; based on 262 attacks of vestibular migraine treated in 124 participants; very low‐certainty evidence). We did not identify any evidence on the change in vertigo using a continuous scale. Only one of the studies assessed serious adverse events. No events were noted in either group, but as the sample size was small we cannot be sure if there are risks associated with taking triptans for this condition (0/75 receiving triptans, 0/39 receiving placebo; 1 study; 114 participants; very low‐certainty evidence).

**Authors' conclusions:**

The evidence for interventions used to treat acute attacks of vestibular migraine is very sparse. We identified only two studies, both of which assessed the use of triptans. We rated all the evidence as very low‐certainty, meaning that we have little confidence in the effect estimates and cannot be sure if triptans have any effect on the symptoms of vestibular migraine. Although we identified sparse information on potential harms of treatment in this review, the use of triptans for other conditions (such as headache migraine) is known to be associated with some adverse effects.

We did not identify any placebo‐controlled randomised trials for other interventions that may be used for this condition. Further research is needed to identify whether any interventions help to improve the symptoms of vestibular migraine attacks and to determine if there are side effects associated with their use.

## Plain language summary

**How effective are medicines used to treat attacks of vestibular migraine?**

**Key messages**

It is not clear whether any medications are effective at treating attacks of vestibular migraine.

There are few studies that have assessed the possible benefits and harms of taking medication at the time of an attack. The available studies are small and the results are inconclusive.

Further work is needed in this area to help establish whether there are any treatments that may be used to help relieve the symptoms of a vestibular migraine attack.

**What is vestibular migraine?**

Migraine (sometimes known as 'classical migraine') is a common condition that causes recurrent headaches. Vestibular migraine is a related condition where the main symptoms are recurring episodes of severe dizziness or vertigo (a spinning sensation). These episodes are often associated with headache, or other migraine‐like symptoms (such as sensitivity to light or sound, nausea and vomiting). It is a relatively common condition, which affects up to 1 in every 100 people, and can have severe effects on day‐to‐day life.

**How is vestibular migraine treated?**

Key approaches to treatment include trying to prevent attacks from starting (preventative treatment), as well as treatments to relieve the symptoms of an attack. There are no widely recommended treatments to manage the symptoms of a vestibular migraine attack. People are sometimes advised to take medicines used to treat headache migraine. The assumption is that these medicines may also work for vestibular migraine.

**What did we want to find out?**

We wanted to find out:

‐ whether there was evidence that any medications work to relieve or stop attacks of vestibular migraine;

‐ whether the treatments might cause any harm.

**What did we do?**

We searched for studies in adults that compared different medications to either no treatment or placebo (dummy) treatment. We used standard methods to assess the quality of the evidence. We rated our confidence in the evidence, based on factors such as study methods, the number of participants in them and the consistency of findings across studies.

**What did we find?**

We found two studies, which included a total of 133 people (75% female). Both studies looked at a specific type of medication known as triptans. The evidence showed that taking a triptan may make very little difference to the number of people whose vertigo symptoms improved. However, there are problems with the evidence, which means that we are uncertain about this result.

One of the studies looked at side effects related to this medication and found that nobody developed serious side effects. However, as this study only included 114 people, it was too small to assess this properly. Therefore, we are not sure whether there may be a risk of harms from taking these treatments.

**What are the limitations of the evidence?**

We have very little confidence in the evidence because the studies conducted were small. There were also some problems with the conduct of the studies, which means that the results may be unreliable. We know that triptans may cause some side effects in people who use them for headache migraine, but we were not able to find out if these side effects are also common when the medications are used for vestibular migraine.

**How up‐to‐date is this evidence?**

This evidence is up‐to‐date to September 2022.

## Summary of findings

**Summary of findings 1:** Triptans compared to placebo for acute attacks of vestibular migraine

**Triptans compared to placebo for acute attacks of vestibular migraine**						
**Patient or population:** adults with acute attacks of vestibular migraine **Setting: **outpatient **Intervention:** triptans **Comparison:** placebo						
**Outcomes**	Anticipated absolute effects^*^ (95% CI)		**Relative effect (95% CI)**	**№ of participants (studies)**	**Certainty of the evidence (GRADE)**	**Comments**
	**Risk with placebo**	**Risk with triptans**				
Improvement in vertigo (global score) ‐ up to 2 hours Assessed with: change from moderate/severe to mild/no vertigo	Study population		RR 0.84 (0.66 to 1.07)	262 (2 RCTs)	⊕⊝⊝⊝ Very low^1,2,3^	Triptans may have little or no effect on improvement in vertigo at up to 2 hours, but the evidence is very uncertain.
	545 improved per 1000 attacks	457 improved per 1000 attacks (359 to 583)				
Change in vertigo	No studies reported this outcome.					
Serious adverse events	Study population		Not estimable	114 (1 RCT)	⊕⊝⊝⊝ Very low^1,3,4^	The evidence is very uncertain about the effect of triptans on serious adverse events.
	0 per 1000	0 per 1000				
*The risk in the intervention group (and its 95% confidence interval) is based on the assumed risk in the comparison group and the **relative effect** of the intervention (and its 95% CI). **CI:** confidence interval; **RCT:** randomised controlled trial; **RR:** risk ratio						
**GRADE Working Group grades of evidence** **High certainty:** we are very confident that the true effect lies close to that of the estimate of the effect. **Moderate certainty:** we are moderately confident in the effect estimate: the true effect is likely to be close to the estimate of the effect, but there is a possibility that it is substantially different. **Low certainty:** our confidence in the effect estimate is limited: the true effect may be substantially different from the estimate of the effect. **Very low certainty:** we have very little confidence in the effect estimate: the true effect is likely to be substantially different from the estimate of effect.						

^1^Serious risk of attrition bias. Multiple domains rated at unclear risk of bias. ^2^Analysis method fails to account for correlation between outcomes reported by the same individual. ^3^Sample size fails to meet the optimal information size, taken to be 300 events for a dichotomous outcome or 400 participants for a continuous outcome, as a rule of thumb. ^4^No events in either arm, therefore cannot calculate an effect estimate.

## Background

### Description of the condition

Vestibular migraine is a form of migraine in which a prominent symptom, often *the* predominant symptom, is recurrent attacks of vertigo (Dieterich 1999; Lempert 2009). These episodes of vertigo are associated with other headache migraine features, such as headache or sensitivity to light or sound. 

The diagnosis of vestibular migraine is challenging because of the overlap of some symptoms with both other balance disorders (such as Ménière's disease) and with headache migraine. People suffering from headache migraine may experience occasional vestibular symptoms, but this does not amount to a diagnosis of 'vestibular migraine'. 

There is now an agreed international classification system that includes categories for 'definite' and 'probable' vestibular migraine (Lempert 2012; described in Appendix 1). In brief, a definite diagnosis of vestibular migraine requires at least five episodes of vestibular symptoms (of moderate to severe intensity) lasting between five minutes and 72 hours. At least half of the episodes must be associated with migrainous features (such as headache, photophobia, phonophobia or a visual aura) and individuals must also have a history of migraine. A diagnosis of 'probable' vestibular migraine requires similar features, but individuals have either migrainous features or a history of migraines (both are not required). Prior to this internationally agreed classification, the criteria proposed by Neuhauser and colleagues were widely used to identify people with vestibular migraine (Neuhauser 2001, see Appendix 2). There is a great deal of similarity between these classification systems, although the Neuhauser criteria do not require a certain number of episodes, or duration of episodes, to make the diagnosis. 

Vestibular migraine is the most common cause of recurrent spontaneous vertigo in adults (Dieterich 2016). The lifetime prevalence of vestibular migraine has been estimated at just under 1% (Neuhauser 2006) and, as such, it is much more common than Ménière's disease. A significant number of cases may still go undiagnosed because of unfamiliarity with the condition or the diagnostic criteria. The disorder may have a slight female preponderance (Lempert 2009). As with many migraine disorders, a genetic susceptibility has been described and candidate genes have been suggested (Frejo 2016).

The pathophysiology of vestibular migraine is still uncertain, but it seems likely to involve similar mechanisms to those of headache migraine. These include activation of the trigeminovascular system (TGVS), which receives nociceptive signals from the large intracranial vessels and the dura (Bernstein 2012). Activation of the TGVS results in neuronal stimulation within parts of the brain involved in pain perception and sensory processing (including the thalamus and the periaqueductal grey) and also causes the release of vasoactive neuropeptides, such as calcitonin gene‐related peptide (CGRP). These, in turn, cause dilatation of the meningeal vessels, extravasation of fluid from the vasculature and release of other inflammatory substances in the dura (Pietrobon 2003), creating a cycle of nerve stimulation. Cortical hyperexcitability, and subsequent cortical spreading depolarisation, also occurs. This may account for the aura or visual symptoms experienced by many migraineurs (Hadjikhani 2001). There may be overlap between headache migraine pathways and those of the vestibular system, accounting for the balance symptoms. For example, the trigeminovascular system receives pain signals from nerves of the dura mater and large intracranial blood vessels, but also from vessels of the inner ear (Vass 1998). Abnormal thalamic activation in response to vestibular stimulation has also been identified in patients with vestibular migraine (Russo 2014). CGRP itself is implicated in vestibular migraine, along with headache migraine, and increased CGRP levels have been linked to the development of symptoms in migraine (Villalón 2009). Work is ongoing into the relevance of CGRP in vestibular migraine, and whether pharmacological targeting of this molecule and its receptors will affect the condition. 

The consequences of vestibular migraine for the individual may be considerable. The unpredictable, disabling attacks of spinning sensory disorientation can be distressing and debilitating in equal measure. This has a considerable impact on engagement with day‐to‐day activities and overall quality of life. 

### Description of the intervention

Current pharmacological treatments for people with vestibular migraine may be prophylactic, or used to treat an acute attack. Many are based on interventions that have been widely used to treat headache migraine. This review is focused on pharmacological interventions that are taken to relieve an attack.  

A variety of pharmacological interventions have been used, or proposed, for treatment of vestibular migraine symptoms. These may be administered by various routes including oral, sublingual, intranasal, intramuscular or subcutaneous injection.  Interventions include the following:

- triptans;
- ergot alkaloids;
- dopamine antagonists;
- antihistamines;
- gepants (CGRP receptor antagonists);
- magnesium;
- 5‐hydroxytryptamine (HT) 3 receptor antagonists;
- caffeine;
- paracetamol;
- non‐steroidal anti‐inflammatory drugs (NSAIDs).

### How the intervention might work

For many of these interventions, the precise mechanism of action is uncertain. However, the use of these medications for headache migraine attacks has resulted in their uptake for vestibular migraine. Interventions may target the underlying processes that are thought to trigger vestibular migraine, or treat the associated symptoms, including headache, nausea and vomiting.

Triptans are commonly used as a treatment for headache migraine. They act as agonists at serotonergic 5HT1B/D receptors. Their effects include vasoconstriction of intracranial vessels, as well as altering the release of other neurotransmitters, which may interrupt the early pathways involved in a migraine attack (Tepper 2002).

Ergot alkaloids (such as dihydroergotamine or ergotamine) were the first specific anti‐migraine therapy available. Ergot alkaloids target serotonergic receptors, but unlike triptans they also affect dopamine and norepinephrine receptors (reviewed in Tfelt‐Hansen 2000 and Bigal 2003). With the emergence of the triptans, ergot alkaloids are used less often in the treatment of migraine.

Dopamine antagonists have been suggested to have some efficacy in treating acute attacks of migraine, but it is not clear whether this is dependent on their effects on dopamine receptors, or due to some other mechanism (reviewed in Akerman 2007).

Antihistamines have been used for their well‐known antiemetic and anti‐vertiginous properties. They are widely used for management of acute vestibular disorders (Hunter 2022). 5‐HT3 receptor antagonists are also primarily used for their anti‐emetic effects. 

CGRP is a neurotransmitter found in numerous locations within the central nervous system and peripheral sensory nerves. Levels of this neurotransmitter have been found to be elevated during headache migraine episodes (Goadsby 1990), and to decrease with the use of triptans (Goadsby 1993). Gepants are novel molecules that target the calcitonin gene‐related peptide receptor, and are increasingly used for headache migraine (reviewed in Moreno‐Ajona 2020).

Magnesium has also been suggested to be of benefit in acute headache migraine episodes (Bigal 2002), although the mechanism of action is unclear.

A variety of analgesics have been used for migraine attacks, including paracetamol and NSAIDs. They may be effective for headache symptoms, but it is uncertain whether they have any benefit for vestibular symptoms. Caffeine is sometimes used in conjunction with analgesics to promote pain relief (Derry 2014a). As well as their analgesic properties, NSAIDs may have additional effects on the underlying pathophysiology of migraine (reviewed in Pardutz 2010).

### Why it is important to do this review

Balance disorders can be difficult to diagnose and treat. There are few specific diagnostic tests, a variety of related disorders and a limited number of interventions that are known to be effective. To determine which topics within this area should be addressed with new or updated systematic reviews, we conducted a scoping and prioritisation process, involving stakeholders (https://ent.cochrane.org/balance-disorders-ent). Vestibular migraine was ranked as one of the highest priority topics during this process (along with persistent postural‐perceptual dizziness and Ménière's disease). 

The impact of vestibular migraine is considerable, with 40% of sufferers reporting sickness from work, and over 70% reporting the impact of their symptoms on daily activities as either moderate or severe (Neuhauser 2006). At present, there are no national or international guidelines to inform the management of this condition, therefore up‐to‐date, reliable evidence syntheses are required to help patients and healthcare professionals determine the benefits and harms of different interventions used for the condition. 

## Objectives

To assess the benefits and harms of pharmacological interventions used to relieve acute attacks of vestibular migraine. 

## Methods

### Criteria for considering studies for this review

#### Types of studies

We included randomised controlled trials (RCTs) and quasi‐randomised trials (where trials were designed as RCTs, but the sequence generation for allocation of treatment used methods such as alternate allocation, birth dates etc). 

We planned to include cross‐over RCTs or cluster‐RCTs, providing we could appropriately account for the clustering in the data analysis (according to methods described in the *Cochrane Handbook for Systematic Reviews of Interventions*) (Handbook 2021, also see Unit of analysis issues).

#### Types of participants

We included studies that recruited participants with a diagnosis of either definite or probable vestibular migraine, according to the International Headache Society (IHS) and Bárány Society criteria (see Appendix 1). We also included studies that used other, established criteria, for example those of Neuhauser 2001 (see Appendix 2). 

If studies had recruited participants with a variety of diagnoses (e.g. vestibular migraine and headache migraine) we planned to include the study if either:

- the majority of participants (≥ 90%) had a diagnosis of vestibular migraine; or
- subgroup data were available that allowed us to identify data relevant specifically to those with vestibular migraine. 

However, we did not identify any studies that this applied to ‐ both included studies specifically recruited individuals with a diagnosis of vestibular migraine. 

#### Types of interventions

We included the following interventions:

- triptans;
- ergot alkaloids;
- dopamine antagonists;
- antihistamines;
- 5‐HT3 receptor antagonists;
- gepants (CGRP receptor antagonists);
- magnesium;
- paracetamol;
- non‐steroidal anti‐inflammatory drugs (NSAIDs).

Caffeine is frequently used in combination with analgesics. If we had identified studies where a combination of an analgesic and caffeine were used then we planned to include these as part of the interventions listed above, and to explore whether there may be additional effects from the caffeine using subgroup analysis. However, this was not necessary. 

The main comparisons were planned to be:

- triptans versus no intervention/placebo;
- ergot alkaloids versus no intervention/placebo;
- dopamine antagonists versus no intervention/placebo;
- antihistamines versus no intervention/placebo;
- gepants versus no intervention/placebo;
- magnesium versus no intervention/placebo;
- 5‐HT3 receptor antagonists versus no intervention/placebo;
- paracetamol versus no intervention/placebo;
- non‐steroidal anti‐inflammatory drugs (NSAIDs) versus no intervention/placebo.

##### Concurrent treatments

There were no limits on the type of concurrent treatments used, providing these were used equally in each arm of the study. We planned to pool studies that included concurrent treatments with those where participants did not receive concurrent treatment, but to conduct subgroup analysis to determine whether the effect estimates may be different in those receiving additional treatment. Again, this was not necessary, as neither study used any concurrent treatments. 

#### Types of outcome measures

We assessed outcomes at the following time points:

- up to 2 hours;
- > 2 to 12 hours;
- > 12 to 72 hours.

An exception was adverse event data, where we used the longest time period of follow‐up. 

We searched the COMET database for existing core outcome sets of relevance to vestibular migraine and vertigo, but were unable to find any published core outcome sets. We therefore conducted a survey of individuals with experience of (or an interest in) balance disorders to help identify the outcomes that should be prioritised. This online survey was conducted with the support of the Ménière's Society and the Migraine Trust, and included 324 participants, who provided information regarding priority outcomes. The review author team used the results of this survey to inform the choice of outcome measures in this review. 

We analysed the following outcomes in the review, but we did not use them as a basis for including or excluding studies.

##### Primary outcomes

- Improvement in vertigo
  - Measured as a dichotomous outcome (improved/not improved), according to self‐report, or according to a change of a specified score (as described by the study authors) on a vertigo rating scale.
- Change in vertigo
  - Measured as a continuous outcome, to identify the extent of change in vertigo symptoms.
- Serious adverse events
  - Including any event that caused death, was life‐threatening, required hospitalisation, resulted in disability or permanent damage, or in congenital abnormality. Measured as the number of participants who experienced at least one serious adverse event during the follow‐up period.

##### Secondary outcomes

- Disease‐specific health‐related quality of life
  - Measured with the Dizziness Handicap Inventory (DHI, Jacobsen 1990), a validated measurement scale in widespread use. If data from the DHI were unavailable we planned to extract data from alternative validated measurement scales, according to the order of preference described in the list below (based on the validity of the scales for this outcome):
    - DHI short form (Tesio 1999);
    - DHI screening tool (Jacobsen 1998).
  - Measured with tools to assess migraine‐related quality of life, such as the Migraine‐Specific Quality of Life Questionnaire (Jhingran 1998).
- Improvement in headache 
  - Measured as a dichotomous outcome (improved/not improved), according to self‐report, or according to a change of specified score (as described by the study authors) on a headache rating scale.
- Improvement in other migrainous symptoms 
  - Measured as a dichotomous outcome (improved/not improved), according to self‐report, or according to a change of specified score (as described by the study authors) on a rating scale.
  - Including nausea and vomiting, photophobia and phonophobia, visual aura.
- Other adverse effects
  - Measured as the number of participants who experienced at least one episode of the specified adverse events during the follow‐up period.
  - Including the following specified adverse effects:
    - gastrointestinal disturbance (e.g. nausea, vomiting, abdominal pain);
    - sleep disturbance (drowsiness, tiredness or problems sleeping);
    - cardiovascular side effects (e.g. palpitations, chest pain or tightness);
    - paraesthesia, flushing, warm or hot sensations;
    - headache.

### Search methods for identification of studies

The Cochrane ENT Information Specialist conducted systematic searches for randomised controlled trials and controlled clinical trials. There were no language, publication year or publication status restrictions. The date of the search was 23 September 2022.

#### Electronic searches

The Information Specialist searched:

- the Cochrane ENT Trials Register (searched via the Cochrane Register of Studies to 23 September 2022);
- the Cochrane Central Register of Controlled Trials (CENTRAL) (searched via the Cochrane Register of Studies to 23 September 2022);
- Ovid MEDLINE(R) Epub Ahead of Print, In‐Process & Other Non‐Indexed Citations, Ovid MEDLINE(R) Daily and Ovid MEDLINE(R) (1946 to 23 September 2022);
- Ovid Embase (1974 to 23 September 2022);
- Web of Knowledge, Web of Science (1945 to 23 September 2022);
- ClinicalTrials.gov, www.clinicaltrials.gov (to 23 September 2022);
- World Health Organization (WHO) International Clinical Trials Registry Platform (ICTRP), https://trialsearch.who.int/ (to 23 September 2022).

The Information Specialist modelled subject strategies for databases on the search strategy designed for CENTRAL. The strategy has been designed to identify all relevant studies for a suite of reviews on various interventions for vestibular migraine. Where appropriate, they were combined with subject strategy adaptations of the highly sensitive search strategy designed by Cochrane for identifying randomised controlled trials and controlled clinical trials (as described in the Technical Supplement to Chapter 4 of the *Cochrane Handbook for Systematic Reviews of Interventions* version 6.1) (Lefebvre 2020). Search strategies for major databases including CENTRAL are provided in Appendix 3.

#### Searching other resources

We scanned the reference lists of identified publications for additional trials and contacted trial authors if necessary. In addition, the Information Specialist searched Ovid MEDLINE to retrieve existing systematic reviews relevant to this systematic review, so that we could scan their reference lists for additional trials. The Information Specialist also ran non‐systematic searches of Google Scholar to identify trials not published in mainstream journals.

We did not perform a separate search for adverse effects. We considered adverse effects described in included studies only.

### Data collection and analysis

#### Selection of studies

At least two review authors or coworkers (of SC, AD, KG, LHK, KW) independently screened the titles and abstracts using Covidence to identify studies that may be relevant for this review. Any discrepancies were resolved by consensus, or by retrieving the full text of the study for further assessment. 

We obtained the full text for any study that may have been relevant and two authors (of AD, KG, LHK, KW) again independently checked this to determine whether it met the inclusion criteria for the review. Any differences were resolved by discussion and consensus, or through recourse to a third author if necessary. 

We have listed as excluded any studies that were retrieved in full text but subsequently deemed to be inappropriate for the review (according to the inclusion/exclusion criteria), according to the main reason for exclusion. 

The unit of interest for the review was the study, therefore multiple papers or reports of a single study were planned to be grouped together under a single reference identification. We recorded the study selection process in sufficient detail to complete a PRISMA flow diagram (see Figure 1) and the Characteristics of excluded studies table. 

**1:**
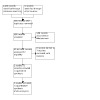
Flow chart of study retrieval and selection.

##### Screening eligible studies for trustworthiness

We assessed all studies meeting our inclusion criteria for trustworthiness using a screening tool developed by Cochrane Pregnancy and Childbirth. This tool includes specified criteria to identify studies that are considered sufficiently trustworthy to be included in the review (see Appendix 4). If any studies were assessed as being potentially 'high risk', we planned to contact the study authors to obtain further information or address any concerns. We planned to exclude 'high risk' studies from the main analyses of the review if we were unable to contact the authors, or there was persisting uncertainty about the study, and only include studies with concerns as part of a sensitivity analysis (see Sensitivity analysis). The process is outlined in Figure 2.

**2:**
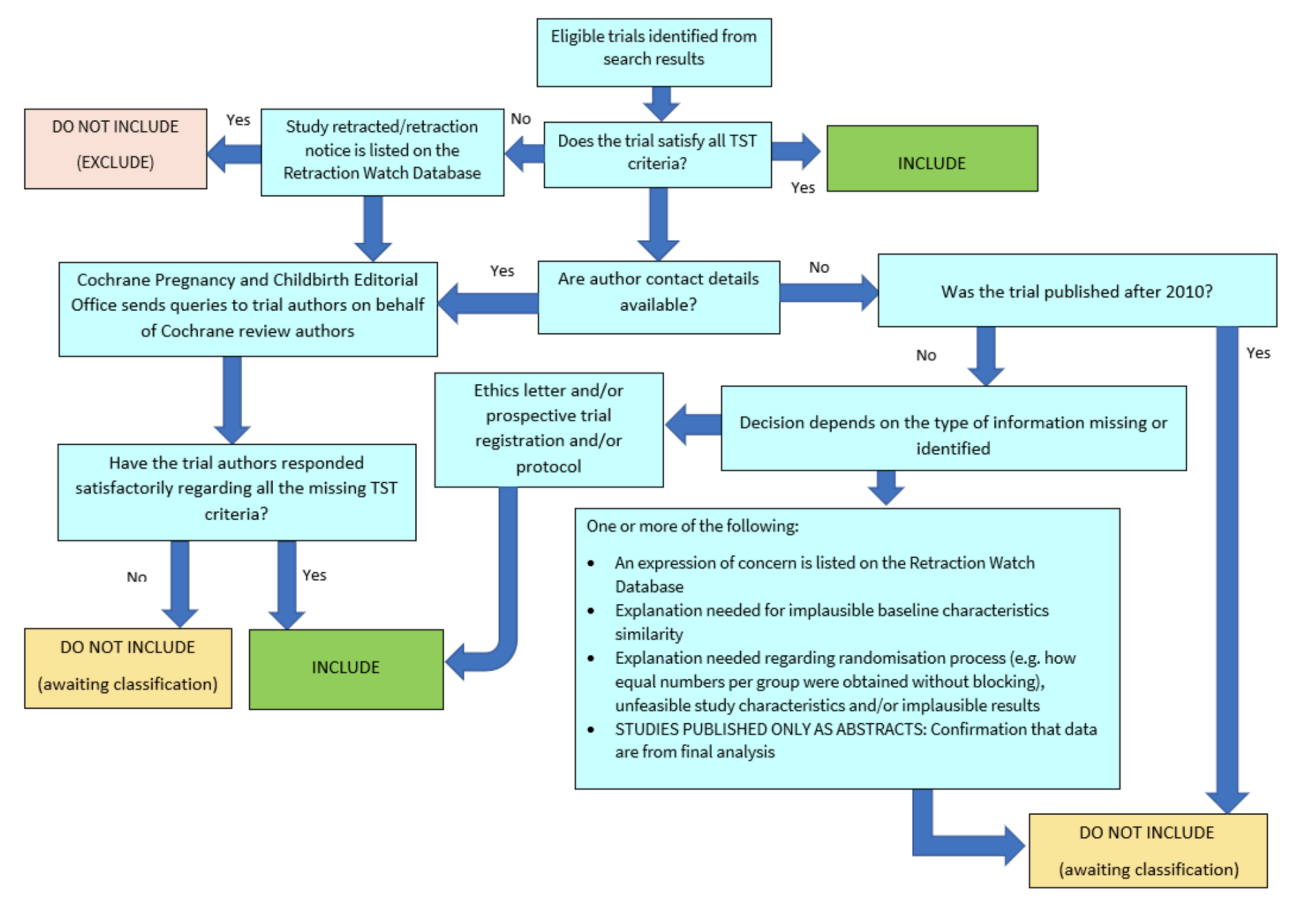
The Cochrane Pregnancy and Childbirth Trustworthiness Screening Tool

The studies included in this review were not considered to be at high risk when using the trustworthiness tool. We did not identify any additional studies that we excluded on this basis.

#### Data extraction and management

At least two review authors (of AD, LHK, KW) independently extracted outcome data from each study using a standardised data collection form. Any discrepancies in the data extracted by the two authors was checked against the original reports, and differences were resolved through discussion and consensus, with recourse to a third author where necessary. 

We included key characteristics of the studies, including the following information:

- study design, duration of the study, number of study centres and location, study setting and dates of the study;
- information on the participants, including the number randomised, those lost to follow‐up or withdrawn, the number analysed, the age of participants, gender, features of the condition (e.g. probable or definite vestibular migraine), diagnostic criteria used, inclusion and exclusion criteria for the individual studies;
- details of the intervention, comparator, and concomitant treatments or excluded medications;
- the outcomes specified and reported by the study authors, including the time points;
- funding for the study and any conflicts of interest for the study authors;
- information required to assess the risk of bias in the study, and to enable GRADE assessment of the evidence.

Once the extracted data were checked and any discrepancies were resolved, a single author (KW) transferred the information to Review Manager 5 (RevMan 2020). 

The primary effect of interest for this review was the effect of treatment assignment (which reflects the outcomes of treatment for people who were assigned to the intervention) rather than a per protocol analysis (the outcomes of treatment only for those who completed the full course of treatment as planned). For the outcomes of interest in this review, we extracted the findings from the studies on an available case basis, i.e. all available data from all participants at each time point, based on the treatment to which they were randomised. This was irrespective of adherence, or whether participants had received the intervention as planned.

In addition to extracting pre‐specified information about study characteristics and aspects of methodology relevant to risk of bias, we extracted the following summary statistics for each study and outcome:

- For binary data: we extracted information on the number of participants experiencing an event, and the number of participants assessed at that time point.
- For ordinal scale data: some data were collected by the study authors with an ordinal scale (a symptom rating scale of 0 to 3). However, the authors of both studies presented the results as binary data (improved/not improved), therefore we extracted the results as above.
- We did not identify any continuous data or time‐to‐event data for this review. 

If necessary, we converted data found in the studies to a format appropriate for meta‐analysis, according to the methods described in the *Cochrane Handbook for Systematic Reviews of Interventions* (Handbook 2021). 

We pre‐specified time points of interest for the outcomes in this review. Where studies reported data at multiple time points, we took the longest available follow‐up point within each of the specific time frames. For example, if a study reported an outcome at three hours and five hours of follow‐up then the five‐hour data would have been included for the time point more than two to six hours.

#### Assessment of risk of bias in included studies

Two authors (of AD, LHK, KW) undertook assessment of the risk of bias of the included studies independently, with the following taken into consideration, as guided by the *Cochrane Handbook for Systematic Reviews of Interventions* (Handbook 2011).

- sequence generation;
- allocation concealment;
- blinding;
- incomplete outcome data;
- selective outcome reporting; and
- other sources of bias.

We used the Cochrane risk of bias tool (Handbook 2011), which involves describing each of these domains as reported in the study and then assigning a judgement about the adequacy of each entry: 'low', 'high' or 'unclear' risk of bias.

#### Measures of treatment effect

We summarised the effects of binary outcomes (e.g. serious adverse effects) as risk ratios (RR) with 95% confidence intervals (CIs). For the key outcomes that we present in the summary of findings tables, we have also expressed the results as absolute numbers based on the pooled results, and compared to the assumed risk. 

#### Unit of analysis issues

The studies included in this review both had unit of analysis issues. The first study involved participants taking medication up to three times (for three separate attacks of vestibular migraine) over the course of the study (NCT02447991). The response to treatment was recorded for each attack. This led to two concerns with the data. Firstly, the data from different attacks in the same individual are not independent ‐ an individual who responds to treatment for one attack may be more likely to respond again. Secondly, individuals experienced different numbers of attacks over the course of the study. A participant who experienced just one attack would only contribute one data point to the results, whilst an individual who experienced three attacks would contribute three (correlated) data points. This skews the overall result towards the outcome for people who suffered more frequent attacks, which may not be relevant for the whole population of people with vestibular migraine. 

The second study was a cross‐over trial (Neuhauser 2003). Data were reported as the response to treatment per attack, and this study included a total of 17 attacks experienced by 10 participants. Attacks experienced by the same individual cannot be regarded as independent. Ideally, results from this study would be analysed using a paired analysis (where each participant acts as their own control) according to the methods stated in the Handbook 2021. However, insufficient data were reported to allow this. We have therefore analysed the data as if they were independent, but acknowledge that this may lead to some inaccuracy in the results.

We took advice from the Cochrane Methods Support Unit regarding these issues. Given that the data available for this review were sparse, they considered it acceptable to include these data in the review, but highlight the limitations of the analysis, and take this into account when using GRADE to assess the certainty of the analysis. 

#### Dealing with missing data

We planned to contact study authors via email whenever the outcome of interest was not reported, if the methods of the study suggested that the outcome had been measured. We did the same if not all data required for meta‐analysis were reported (for example, standard deviations), unless we were able to calculate them from other data reported by the study authors. 

#### Assessment of heterogeneity

We assessed clinical heterogeneity by examining the included studies for potential differences between them in the types of participants recruited, interventions or controls used and the outcomes measured.

We used the I^2^ statistic to quantify inconsistency among the studies in each analysis. We also considered the P value from the Chi^2^ test. 

#### Assessment of reporting biases

We assessed reporting bias as within‐study outcome reporting bias and between‐study publication bias.

##### Outcome reporting bias (within‐study reporting bias)

We assessed within‐study reporting bias by comparing the outcomes reported in the published report against the study protocol or trial registry, whenever this could be obtained. If the protocol or trial registry entry was not available, we compared the outcomes reported to those listed in the methods section. If results are mentioned but not reported adequately in a way that allows analysis (e.g. the report only mentions whether the results were statistically significant or not), bias in a meta‐analysis is likely to occur. We then sought further information from the study authors. If no further information could be found, we noted this as being a 'high' risk of bias with the risk of bias tool. If there was insufficient information to judge the risk of bias we noted this as an 'unclear' risk of bias (Handbook 2011). 

##### Publication bias (between‐study reporting bias)

We planned to assess funnel plots to identify the likelihood of unpublished data, however we did not identify sufficient studies to be able to do this. We also planned to report on whether there were any studies identified through trial registries and other sources (Searching other resources), with unpublished reports. However, we did not identify any unpublished trials. 

#### Data synthesis

##### Meta‐analysis of numerical data

Where possible and appropriate (where participants, interventions, comparisons and outcomes were sufficiently similar in the studies identified) we conducted a quantitative synthesis of results, using RevMan 2020. We anticipated that the underlying effect of the intervention may vary between studies, as there were likely to be differences between participants, settings and the interventions used for each study. We therefore used a random‐effects method for meta‐analysis. We explored whether the use of a fixed‐effect model substantially altered the effect estimates (see Sensitivity analysis). 

For dichotomous data, we analysed treatment differences as a risk ratio (RR) calculated using the Mantel‐Haenszel methods.

##### Synthesis using other methods

If we were unable to pool numerical data in a meta‐analysis for one or more outcomes we planned to provide a synthesis of the results using alternative methods, following the guidance in Chapter 12 of the Handbook 2021. However, this was not necessary.

#### Subgroup analysis and investigation of heterogeneity

If statistical heterogeneity was identified for any comparisons, we planned to assess this considering the following subgroups:

- Different types of intervention, within a specific group.
- Use of any concomitant treatment.
- Diagnosis of vestibular migraine.
- Age of the participants.
- Sex of the participants.

However, due to the paucity of data available, and the few meta‐analyses included in this review, we did not carry out any subgroup analysis. 

For the outcome 'improvement of other migrainous symptoms' we planned to pool different symptoms (such as nausea and vomiting, photophobia and phonophobia, visual aura) and assess this as a composite measure. However, as we only identified a single study that assessed these outcomes, we have presented them as separate measures. 

#### Sensitivity analysis

As few studies were identified for meta‐analysis, the random‐effects model may provide an inaccurate measure of the between‐studies variance. Therefore, we explored the impact of using a fixed‐effect model using a sensitivity analysis. 

If there was uncertainty over the diagnostic criteria used for participants in the studies (for example, if it was not clear whether participants were diagnosed using criteria analogous to the IHS‐Bárány Society criteria) then we planned to explore this by including/excluding those studies from the analysis. However, this was not necessary. 

We used the Cochrane Pregnancy and Childbirth Screening Tool to identify any studies where there were concerns over the data available. We planned to exclude any studies that we identified as high risk with this tool from the main analyses in the review, but we intended to explore the impact of including the data from these studies through a sensitivity analysis. Again, as we did not exclude any studies on the basis of this tool, no sensitivity analysis was required. 

#### Summary of findings and assessment of the certainty of the evidence

Two independent authors (KG, KW) used the GRADE approach to rate the overall certainty of evidence using GRADEpro GDT (https://gradepro.org/) and the guidance in Chapter 14 of the *Cochrane Handbook for Systematic Reviews of Interventions* (Handbook 2021). Disagreements were resolved through discussion and consensus. The certainty of evidence reflects the extent to which we are confident that an estimate of effect is correct, and we applied this in the interpretation of results. There are four possible ratings: high, moderate, low and very low. A rating of high certainty of evidence implies that we are confident in our estimate of effect and that further research is very unlikely to change our confidence in the estimate of effect. A rating of very low certainty implies that any estimate of effect obtained is very uncertain.

The GRADE approach rates evidence from RCTs that do not have serious limitations as high certainty. However, several factors can lead to the downgrading of the evidence to moderate, low or very low. The degree of downgrading is determined by the seriousness of these factors:

- Study limitations (risk of bias):
  - This was assessed using the rating from the Cochrane risk of bias tool for the study or studies included in the analysis. We rated down either one or two levels, depending on the number of domains that had been rated at high or unclear risk of bias. 
- Inconsistency:
  - This was assessed using the I^2^ statistic and the P value for heterogeneity for all meta‐analyses, as well as by visual inspection of the forest plot. For results based on a single study we rated this domain as no serious inconsistency.
- Indirectness of evidence:
  - We took into account whether there were concerns over the population included in the study or studies for each outcome, as well as whether additional treatments were offered that may impact on the efficacy of the intervention under consideration. 
- Imprecision:
  - We took into account the sample size and the width of the confidence interval for each outcome. If the sample size did not meet the optimal information size (i.e. < 400 people for continuous outcomes or < 300 events for dichotomous outcomes), or the confidence interval crossed the small effect threshold we rated down one level. If the sample size did not meet the optimal information size and the confidence interval included both potential harm and potential benefit we rated down twice. We also rated down twice for very tiny studies (e.g. 10 to 15 participants in each arm), regardless of the estimated confidence interval.
- Publication bias:
  - We considered whether there were likely to be unpublished studies that may impact on our confidence in the results obtained. 

We used a minimally contextualised approach, and rated the certainty in the interventions having an important effect (Zeng 2021). Where possible, we used agreed minimally important differences (MIDs) for continuous outcomes as the threshold for an important difference. Where no MID was identified, we provide an assumed MID based on agreement between the authors. For dichotomous outcomes, we looked at the absolute effects when rating imprecision, but also took into consideration the GRADE default approach (rating down when a RR crosses 1.25 or 0.80). We have justified all decisions to downgrade the certainty of the evidence using footnotes, and added comments to aid the interpretation of the findings, where necessary. 

We prepared a separate summary of findings table for the main comparison:

- triptans versus no intervention/placebo.

We included all primary outcomes in the summary of findings table. We prioritised outcomes at the time point 'up to two hours' for presentation in the table. We have also included a full GRADE profile for all results (Table 2).

**1:** GRADE profile: Triptans versus placebo for acute attacks of vestibular migraine

**Certainty assessment**							**№ of participants**		**Effect**		**Certainty**
**№ of studies**	**Study design**	**Risk of bias**	**Inconsistency**	**Indirectness**	**Imprecision**	**Other considerations**	**Triptans**	**Placebo**	**Relative** **(95% CI)**	**Absolute** **(95% CI)**	
**Improvement in vertigo (global score): up to 2 hours (assessed with: change from moderate/severe to mild/no vertigo)**											
2	Randomised trials	Very serious^a,b^	Not serious	Not serious	Serious^c^	None	76/161* (47.2%)	55/101* (54.5%)	**RR 0.84** (0.66 to 1.07)	**87 fewer per 1000** (from 185 fewer to 38 more)	⨁◯◯◯ Very low
**Improvement in vertigo (global score): > 12 hours to 72 hours (assessed with: change from moderate/severe to no/mild vertigo)**											
1	Randomised trials	Very serious^a,b^	Not serious	Not serious	Serious^c^	None	97/109* (89.0%)	54/61* (88.5%)	**RR 1.01** (0.90 to 1.12)	**9 more per 1000** (from 89 fewer to 106 more)	⨁◯◯◯ Very low
**Serious adverse events**											
1	Randomised trials	Serious^a^	Not serious	Not serious	Very serious^c,d^	None	0/75 (0.0%)	0/39 (0.0%)	Not estimable		⨁◯◯◯ Very low
**Improvement in headache: up to 2 hours (assessed with: change from moderate/severe to mild/no headache)**											
2	Randomised trials	Very serious^a,b^	Not serious	Not serious	Serious^c^	None	45/154* (29.2%)	40/94* (42.6%)	**RR 0.69** (0.49 to 0.96)	**132 fewer per 1000** (from 217 fewer to 17 fewer)	⨁◯◯◯ Very low
**Improvement in headache: > 12 hours to 72 hours (assessed with: change from moderate/severe to mild/no headache)**											
1	Randomised trials	Very serious^a,b^	Not serious	Not serious	Serious^c^	None	94/109* (86.2%)	46/62* (74.2%)	**RR 1.16** (0.99 to 1.37)	**119 more per 1000** (from 7 fewer to 275 more)	⨁◯◯◯ Very low
**Improvement in other migrainous symptoms: nausea and vomiting at up to 2 hours (assessed with: change from moderate/severe to mild/no symptoms)**											
1	Randomised trials	Very serious^a,b^	Not serious	Not serious	Serious^c^	None	67/150* (44.7%)	50/89* (56.2%)	**RR 0.80** (0.62 to 1.03)	**112 fewer per 1000** (from 213 fewer to 17 more)	⨁◯◯◯ Very low
**Improvement in other migrainous symptoms: nausea and vomiting at > 12 to 72 hours (assessed with: change from moderate/severe to mild/no symptoms)**											
1	Randomised trials	Very serious^a,b^	Not serious	Not serious	Serious^c^	None	101/108* (93.5%)	57/62* (91.9%)	**RR 1.02** (0.93 to 1.11)	**18 more per 1000** (from 64 fewer to 101 more)	⨁◯◯◯ Very low
**Improvement in other migrainous symptoms: photo‐ and phonophobia at up to 2 hours**											
1	Randomised trials	Very serious^a,b^	Not serious	Not serious	Very serious^c,e^	None	59/151* (39.1%)	33/89* (37.1%)	**RR 1.05** (0.75 to 1.47)	**19 more per 1000** (from 93 fewer to 174 more)	⨁◯◯◯ Very low
**Improvement in other migrainous symptoms: photo‐ and phonophobia at > 12 to 72 hours**											
1	Randomised trials	Very serious^a,b^	Not serious	Not serious	Serious^c^	None	95/109* (87.2%)	45/62* (72.6%)	**RR 1.20** (1.01 to 1.42)	**145 more per 1000** (from 7 more to 305 more)	⨁◯◯◯ Very low
**Other adverse effects: gastrointestinal disturbance**											
1	Randomised trials	Serious^a^	Not serious	Not serious	Very serious^c,e^	None	19/75 (25.3%)	8/39 (20.5%)	**RR 1.24** (0.60 to 2.56)	**49 more per 1000** (from 82 fewer to 320 more)	⨁◯◯◯ Very low
**Other adverse effects: sleep disturbance**											
1	Randomised trials	Serious^a^	Not serious	Not serious	Serious^c^	None	51/75 (68.0%)	11/39 (28.2%)	**RR 2.41** (1.43 to 4.07)	**398 more per 1000** (from 121 more to 866 more)	⨁⨁◯◯ Low
**Other adverse effects: cardiovascular side effects**											
1	Randomised trials	Serious^a^	Not serious	Not serious	Very serious^c,e^	None	5/75 (6.7%)	3/39 (7.7%)	**RR 0.87** (0.22 to 3.44)	**10 fewer per 1000** (from 60 fewer to 188 more)	⨁◯◯◯ Very low
**Other adverse effects: paraesthesia, flushing, warm or hot sensations**											
1	Randomised trials	Serious^a^	Not serious	Not serious	Very serious^c,e^	None	7/75 (9.3%)	5/39 (12.8%)	**RR 0.73** (0.25 to 2.14)	**35 fewer per 1000** (from 96 fewer to 146 more)	⨁◯◯◯ Very low
**Other adverse effects: headache**											
1	Randomised trials	Serious^a^	Not serious	Not serious	Very serious^c,e^	None	13/75 (17.3%)	9/39 (23.1%)	**RR 0.75** (0.35 to 1.60)	**58 fewer per 1000** (from 150 fewer to 138 more)	⨁◯◯◯ Very low

**CI:** confidence interval; **RR:** risk ratio * Numerator and denominator for this analysis are the number of events with improvement, compared to the total number of events (as opposed to the number of participants). ^a^Serious risk of attrition bias. Multiple domains rated at unclear risk of bias. ^b^Analysis method fails to account for correlation between outcomes reported by the same individual. ^c^Sample size fails to meet the optimal information size, taken to be 300 events for a dichotomous outcome or 400 participants for a continuous outcome, as a rule of thumb. ^d^No events in either arm, therefore cannot calculate an effect estimate. ^e^Confidence interval includes the potential for either substantial benefit or substantial harm from the intervention.

## Results

### Description of studies

#### Results of the search

The searches in September 2022 retrieved a total of 1186 records. This reduced to 558 after the removal of duplicates. We screened the titles and abstracts of these 558 records. We discarded 548 records and assessed 10 full‐text records, which were linked to nine studies. 

We excluded seven studies (eight records) with reasons recorded in the review (see Excluded studies). We included two completed studies (two records) where results were available. We did not identify any ongoing studies for this review. 

A flow chart of study retrieval and selection is provided in Figure 1.

#### Included studies

We included two RCTs in this review (NCT02447991; Neuhauser 2003). Details of the individual studies can be found in the Characteristics of included studies. 

##### Study design

Both studies were described as randomised controlled trials and included two arms, comparing an active medication to a placebo. One study used a cross‐over method, where participants received the alternate intervention after their first attack (Neuhauser 2003). The duration of treatment and follow‐up was not reported. Although 19 participants were recruited into this study, only 10 participants experienced a vestibular migraine attack during the course of the study.

The NCT02447991 study aimed to follow up participants until they had experienced three attacks of vestibular migraine (over a period of up to four years). Participants were randomised to use either triptan or placebo, but were asked to treat up to three attacks with the same medication. NCT02447991 was also the largest study, although there was a discrepancy in the reported numbers of participants from two versions of the results (the trial registry states 134 participants; the accompanying statistical analysis plan, which includes the results, states 114 participants).

Further details on the analysis of data from these studies, to account for the cross‐over design and multiple data points from the same individual, are described in Unit of analysis issues, above. 

##### Participants 

Both studies recruited adult participants with a diagnosis of vestibular migraine.

##### Diagnosis of vestibular migraine 

NCT02447991 used the IHS criteria for the diagnosis of definite vestibular migraine (see Appendix 1 for details). Neuhauser 2003 used the criteria for definite vestibular migraine outlined in Neuhauser 2001 (see Appendix 2 for details). 

##### Features of vestibular migraine

Neither of the included studies gave details on the duration of the disease. All participants had to have experienced at least two attacks of vestibular migraine in the 12 months prior to entry to the study, but no additional information was provided on the frequency of attacks at baseline. 

##### Interventions and comparisons 

Both studies evaluated triptans. One evaluated zolmitriptan (Neuhauser 2003) and the other evaluated rizatriptan (NCT02447991). 

##### Comparison 1: Triptans versus placebo

Neuhauser 2003 used 2.5 mg zolmitriptan to be taken orally when symptoms of vestibular migraine were moderate or severe. A second dose of study medication (either triptan or placebo, accordingly) or a rescue medication (dimenhydrinate 150 mg for vertigo and paracetamol 500 mg for headache) could be taken after two hours, if required. NCT02447991 used one capsule of 10 mg rizatriptan to be taken orally during an acute episode. 

##### Outcomes 

###### 1. Improvement in vertigo

This was assessed by both of the studies, with the same scoring system, which appeared to be a global score of vertigo symptoms. Participants in the included studies were asked to rate their vertigo symptoms at the time of an attack using a four‐point scale, where 0 = no symptoms, 1 = mild symptoms, 2 = moderate symptoms and 3 = severe symptoms. The same scale was used to assess the severity of symptoms after receiving treatment. Both studies reported on the number of migraine attacks in which the severity of vertigo symptoms changed from moderate/severe down to mild/no symptoms. 

###### 2. Change in vertigo

Neither study considered the change in vertigo using a continuous scale. 

###### 3. Serious adverse events

Only one of the included studies appeared to systematically assess and report serious adverse events (NCT02447991). 

###### 4. Disease‐specific health‐related quality of life

This outcome was not assessed or reported by either study. 

###### 5. Improvement in headache

Both studies assessed the improvement in headache, using the same four‐point scale that had been used to assess severity of vertigo. 

###### 6. Improvement in other migrainous symptoms 

One study also considered other migrainous symptoms, again using the same four‐point scale, including nausea and vomiting, and photo‐ and phonophobia (NCT02447991). 

###### 7. Other adverse effects

One study reported on a number of adverse effects that were of interest in this review, including gastrointestinal disturbance, sleep disturbance, cardiovascular side effects, paraesthesia/flushing/warm or hot sensations, and headache. 

#### Excluded studies

After assessing the full text, we excluded seven studies (linked to eight records) from this review. The main reason for exclusion for each study is listed below.

Two studies were not randomised controlled trials (ACTRN12616000683437; ChiCTR1800014766).

Two articles were systematic reviews (Byun 2021; Hou 2020). We checked the reference lists of these to ensure that any relevant studies were included in this review. 

The three remaining studies did not consider treatment of acute attacks of vestibular migraine (Furman 2009; Furman 2011; Marcus 2006). Although they were RCTs of people with vestibular migraine, they aimed to assess whether prophylactic treatment with triptans would prevent the onset of motion sickness symptoms (when participants were subjected to a vestibular stimulus). Therefore, the aims, conduct and outcomes reported in these studies were not of relevance to this review.

### Risk of bias in included studies

See Figure 3 for the risk of bias graph (our judgements about each risk of bias item presented as percentages across all included studies) and Figure 4 for the risk of bias summary (our judgements about each risk of bias item for each included study). Both of the studies included in this review received a rating of high risk of bias in at least one domain. 

**3:**
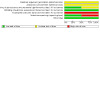
Risk of bias graph (our judgements about each risk of bias item presented as percentages across all included studies).

**4:**
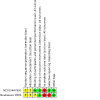
Risk of bias summary (our judgements about each risk of bias item for each included study).

#### Allocation

Neither study provided any information about the generation of the random sequence, or methods used to conceal allocation to each group. Therefore, we rated this domain at unclear risk of bias for both studies. 

#### Blinding

Both studies reported the use of a placebo and indicated that study participants were blinded to their treatment allocation. NCT02447991 also described study personnel as blinded, therefore we rated this at low risk of performance and detection bias. It was unclear whether study personnel in the Neuhauser 2003 study were also blinded. Therefore, we rated this study at unclear risk of performance bias, but low risk of detection bias.  

#### Incomplete outcome data

We rated both studies at high risk of attrition bias. A high level of dropout occurred in the NCT02447991 study and this was not balanced across the two groups (22.2% in the placebo arm, 33.7% in the intervention arm). Although only three participants dropped out of the study Neuhauser 2003, this was a very small study (19 participants in total), therefore even this small number of missing data points may influence the conclusions of this study. 

#### Selective reporting

We rated the NCT02447991 study at low risk of selective reporting bias, as outcomes were reported in accordance with the trial registry details. No protocol or trial registration was available for Neuhauser 2003. In addition, the results of this cross‐over trial were not reported in a way that correctly accounted for the paired nature of the data. We therefore rated this study at high risk of selective reporting bias. 

#### Other potential sources of bias

We did not have any additional concerns regarding the studies. 

### Effects of interventions

See: Table 1

#### Triptans versus placebo for vestibular migraine

##### Improvement in vertigo

Both studies considered the improvement in vertigo using a four‐point scale (0 = no vertigo, 1 = mild vertigo, 2 = moderate vertigo and 3 = severe vertigo). Improvement was considered a reduction from moderate or severe vertigo to mild or no vertigo. Both studies assessed this by considering individual attacks of vertigo, therefore the denominator in the analysis is the total number of attacks, rather than the total number of participants. As described above, this causes a unit of analysis error in the results, which we were unable to account for with the data reported. Therefore, these analyses are shown for completeness, but the results should be interpreted with caution. This is reflected in the GRADE certainty rating for these outcomes. 

###### Improvement in global score

####### At up to 2 hours

The risk ratio for improvement in vertigo at up to two hours with triptans was 0.84 (95% confidence interval (CI) 0.66 to 1.07; 2 studies; 262 events; 124 participants; I^2^ = 0%; Analysis 1.1; very low‐certainty evidence). A sensitivity analysis using a fixed‐effect model caused very little change in this estimate (see Table 3). 

**2:** Sensitivity analyses

**Primary analysis**	**Sensitivity analysis result**	**Description of analysis**
Analysis 1.1 Improvement in vertigo frequency at up to 2 hours	RR 0.85 (95% CI 0.67 to 1.08)	Fixed‐effect model
Analysis 1.2 Improvement in headache at up to 2 hours	RR 0.68 (95% CI 0.49 to 0.96)	Fixed‐effect model

CI: confidence interval; RR: risk ratio

####### At 2 to 12 hours

No results were reported at this duration of follow‐up. 

####### At > 12 to 72 hours

The risk ratio for improvement in vertigo at 24 hours with triptans was 1.01 (95% CI 0.90 to 1.12; 1 study; 170 events; 114 participants; Analysis 1.1; very low‐certainty evidence). 

###### Improvement in vertigo frequency

This outcome was not assessed by either study. 

##### Change in vertigo

This outcome was not assessed by either study. 

##### Serious adverse events

One study assessed serious adverse events (NCT02447991), and reported that no events occurred in either group (1 study; 114 participants; very low‐certainty evidence).

##### Improvement in headache

Both studies also considered an improvement in headache using the same four‐point scale as was used for vertigo. 

###### At up to 2 hours

The risk ratio for improvement in headache at up to two hours with triptans was 0.69 (95% CI 0.49 to 0.96; 2 studies; 248 events; 124 participants; I^2^ = 0%; Analysis 1.2; very low‐certainty evidence).  A sensitivity analysis using a fixed‐effect model caused very little change in this estimate (see Table 3).

###### At 2 to 12 hours

No results were reported at this duration of follow‐up. 

###### At > 12 to 72 hours

The risk ratio for improvement in headache at 24 hours with triptans was 1.16 (95% CI 0.99 to 1.37; 1 study; 171 events; 114 participants; Analysis 1.2; very low‐certainty evidence). This analysis only included events in which participants did not take additional medication over the 24‐hour follow‐up period. 

##### Improvement in other migrainous symptoms

One study assessed this outcome (NCT02447991). Again, a four‐point scale was used to assess improvement in symptoms, as described above. 

###### Improvement in nausea and vomiting

####### At up to 2 hours

The risk ratio for improvement in nausea and vomiting at up to two hours with triptans was 0.80 (95% CI 0.62 to 1.03; 1 study; 239 events; 114 participants; Analysis 1.3; very low‐certainty evidence). 

####### At 2 to 12 hours

No results were reported at this duration of follow‐up. 

####### At > 12 to 72 hours

The risk ratio for improvement in nausea and vomiting at 24 hours with triptans was 1.02 (95% CI 0.93 to 1.11; 1 study; 170 events; 114 participants; Analysis 1.3; very low‐certainty evidence). This analysis only included events in which participants did not take additional medication over the 24‐hour follow‐up period. 

###### Improvement in photophobia and phonophobia

####### At up to 2 hours

The risk ratio for improvement in photophobia and phonophobia at up to two hours with triptans was 1.05 (95% CI 0.75 to 1.47; 1 study; 240 events; 114 participants; Analysis 1.3; very low‐certainty evidence). 

####### At 2 to 12 hours

No results were reported at this duration of follow‐up. 

####### At >12 to 72 hours

The risk ratio for improvement in nausea and vomiting at 24 hours with triptans was 1.20 (95% CI 1.01 to 1.42; 1 study; 171 events; 114 participants;  Analysis 1.3; very low‐certainty evidence). This analysis only included events in which participants did not take additional medication over the 24‐hour follow‐up period. 

##### Other adverse effects

One study reported on the adverse effects that had been pre‐specified as of interest in this review (NCT02447991). 

###### Gastrointestinal disturbance

The risk ratio for gastrointestinal disturbance (nausea, vomiting or stomach upset) with triptans was 1.24 (95% CI 0.60 to 2.56; 1 study; 114 participants; Analysis 1.4; very low‐certainty evidence).

###### Sleep disturbance

The risk ratio for sleep disturbance (sleepiness or drowsiness) with triptans was 2.41 (95% CI 1.43 to 4.07; 1 study; 114 participants; Analysis 1.4; low‐certainty evidence).

###### Cardiovascular side effects

The risk ratio for cardiovascular side effects (including heart rhythm problems or chest pain) with triptans was 1.24 (95% CI 0.60 to 2.56; 1 study; 114 participants; Analysis 1.4; very low‐certainty evidence).

###### Paraesthesia, flushing, warm or hot sensations

No studies reported specifically on flushing, warm or hot sensations. One study reported on weakness of the arms, legs or face or loss of sensation (NCT02447991). We considered that this may include paraesthesia, therefore for completeness we have included the data here. The risk ratio for this outcome was 0.73 (95% CI 0.25 to 2.14; 1 study; 114 participants; Analysis 1.4; very low‐certainty evidence). 

###### Headache

One study reported on the worsening of headache (NCT02447991). The risk ratio for headache worsening with triptans was 0.75 (95% CI 0.35 to 1.60; 1 study; 114 participants; Analysis 1.4; very low‐certainty evidence). 

## Discussion

### Summary of main results

This review included two studies, both of which compared the use of triptans (either rizatriptan or zolmitriptan) to placebo, for the treatment of acute attacks of vestibular migraine. Triptans may make little or no difference to the proportion of people who experience an improvement in their vertigo symptoms at up to two hours, or between 12 and 72 hours, but the evidence was very uncertain. We did not identify any evidence on change in vertigo symptoms using a continuous scale for this review. 

When considering headache symptoms, the evidence was also very uncertain. However, fewer people reported an improvement in headache at up to two hours after taking triptans than those who took a placebo. At 12 to 72 hours there was little or no difference between the two groups. There was also little or no difference in other migrainous symptoms (including nausea and vomiting, photo‐ and phonophobia) at both up to two hours, and 12 to 72 hours, but the evidence was all very uncertain. 

One study assessed the occurrence of serious adverse events, but only reported that no events occurred in either group. This evidence is also very uncertain. When considering other (less serious) adverse effects, triptans may increase the proportion of people who experience sleep disturbance. Results for other side effects were all very uncertain, but indicated little or no difference in the proportion of people who experienced gastrointestinal disturbance, cardiovascular side effects, paraesthesia (including flushing and warm or hot sensations) or headache.

### Overall completeness and applicability of evidence

The only evidence we identified for this review related to a single intervention: triptans. One study considered the use of rizatriptan, and the other considered zolmitriptan, so the evidence available is based on these two drugs alone. 

We did not find any evidence for some of our outcomes of interest, including the change in vertigo (using a continuous score) and disease‐specific health‐related quality of life. We also found very limited information on potential harms associated with the use of triptans. However, when triptans are used in equivalent doses to treat other conditions (such as headache migraine or cluster headache) they have been associated with side effects (Derry 2014b; Tepper 2003). 

We also did not identify any randomised controlled trials (RCTs) that compared other interventions (as listed in Types of interventions) to either placebo or no treatment. Therefore, we do not have any information on the efficacy or harms of any other medications used to treat acute attacks of vestibular migraine. 

Both studies used the same method to assess improvement in symptoms ‐ the change in a four‐point scale of symptom severity was converted to a dichotomous outcome (where "improvement" meant a change from severity of 3 or 2 down to 0 or 1). We could not find any information on whether this scale has been validated for used in this context. However, we note that some people may also regard a change in symptoms from 'severe' to 'moderate' as improvement. More work is needed to identify the best ways to measure symptoms of vestibular migraine, and assess what change in symptoms is meaningful and important to people with this condition. 

For the outcome 'improvement in vertigo', people with 'mild' attacks of vestibular migraine were not included. Only attacks classed as moderate or severe were assessed for improvement. In addition, one of the included studies allowed participants to treat up to three attacks of vestibular migraine with the same medication (NCT02447991). Consequently, people who suffered more frequent attacks of vestibular migraine will have contributed more outcome data to the analysis than those who suffered only one attack over the course of the trial. The overall effect of these study features means that the outcome data may be skewed towards the effect for people with more severe or frequent attacks of vestibular migraine. As a consequence, the results may not be applicable to those with less frequent, or less severe attacks. 

Triptans are widely used to treat headache migraine, where there is stronger evidence of their efficacy (Derry 2014b), in contrast to the results of this review of their use in vestibular migraine. It is unclear whether this is because the underlying pathophysiology of the two conditions may differ, or simply because the studies of vestibular migraine are too small or inadequately designed to detect an effect of triptans. 

### Quality of the evidence

We rated all of the evidence in this review as either very low‐ or very low‐certainty, showing that we have little confidence in the effect estimates. Several factors contributed to this assessment. 

Firstly, the total number of participants in these studies was relatively small, meaning that the calculated effect estimates had wide confidence intervals, leading to imprecision. In addition, the number of participants dropping out during the trials was quite considerable. 

We also had concerns about the analysis methods in both studies. One study was a cross‐over trial, where participants received either the triptan, or a placebo, and then switched to the alternative treatment for their next attack (Neuhauser 2003). Ideally, these data would be analysed as 'paired data', comparing the results on each treatment for an individual person. However, the information reported in the study did not allow us to analyse the data in that way. There were also concerns with the analysis in the second study (NCT02447991), where participants received one medication and used this repeatedly to treat several attacks. We were unable to account for the correlation between data points from the same individual. Therefore, many of the analyses presented in this review must be viewed as approximate. 

### Potential biases in the review process

This review only included studies that compared an active intervention to no treatment or placebo. This may mean that we omitted studies that compared one active treatment to another. However, as there is currently no 'gold standard' treatment for acute attacks of vestibular migraine, we considered that this was an appropriate decision. 

### Agreements and disagreements with other studies or reviews

A recent systematic review evaluated the use of pharmacological (and non‐pharmacological) interventions for both the prophylaxis and acute treatment of vestibular migraine (Smyth 2022). The authors of this review included both randomised and non‐randomised studies, therefore the results are not directly comparable with our own review. However, their conclusions are similar to our own ‐ that the overall evidence base for the treatment of vestibular migraine is of low certainty and that well‐designed clinical trials are required in this area. 

## Authors' conclusions

**Implications for practice**

Currently, we have identified only two randomised controlled trials (RCTs) that assessed interventions used for acute attacks of vestibular migraine. The evidence from these studies shows great uncertainty in the effects of triptans, and limited information on the possibility of harms from these medications. The lack of robust evidence in this area should be recognised by people with vestibular migraine and healthcare professionals, when deciding on possible treatments for this condition. 

**Implications for research**

This review was conducted as part of a suite, which evaluates different interventions for the prophylaxis or acute treatment of vestibular migraine (Webster 2022a; Webster 2022b; Webster 2022c). The conclusions below relate to evidence from across the entire suite:

- There is a paucity of RCTs in this field, where active interventions are compared to no treatment or a placebo. Given the subjective nature of symptoms of vestibular migraine, the fluctuating severity of the condition and the lack of a 'gold standard' treatment, we consider that comparison with a placebo arm is vital to allow conclusions to be drawn on the efficacy and harms of different interventions. 
- Wherever possible, trialists should ensure that participants, study personnel and outcome assessors are appropriately blinded to the intervention, to reduce the risk of performance and detection bias affecting the results of studies. 
- Small, underpowered studies do little to improve the evidence base for these interventions. We would advocate the conduct of large, adequately powered, multicentre trials to ensure that more robust conclusions can be drawn from the study results. In addition, trialists need to be aware that there is considerable attrition over the course of these studies and should be prepared to make additional efforts to improve follow‐up. 
- Future studies should also aim to follow up participants for longer periods of time, to identify whether interventions have lasting effects.
- There needs to be consensus on the appropriate outcomes to measure in trials that evaluate interventions for vestibular migraine, with input from different stakeholders, especially including those with the condition. As well as agreeing the types of outcomes that are important, the methods with which these are measured should be considered, including the use of validated scales to assess more subjective outcomes. This would be best achieved with the development of a core outcome set, analogous to that developed for use in trials of headache migraine (Haywood 2021). 

In addition, if study designs are used that include correlated data (such as cross‐over or cluster designs, or where multiple outcomes may be reported by a single individual), trialists must ensure that results are reported in a way that accounts for this correlation, or the evidence arising from these studies is likely to be at risk of bias. 

## History

Protocol first published: Issue 3, 2022

## Appendices

### Appendix 1. International Headache Society (IHS) and Bárány Society criteria for the diagnosis of vestibular migraine

From Lempert 2012:

**Vestibular migraine**

A. At least five episodes with vestibular symptoms of moderate or severe intensity, lasting five minutes to 72 hours.

B. Current or previous history of migraine with or without aura according to the International Classification of Headache Disorders (ICHD).

C. One or more migraine features with at least 50% of the vestibular episodes:

- headache with at least two of the following characteristics: one sided location, pulsating quality, moderate or severe pain intensity, aggravation by routine physical activity;
- photophobia and phonophobia;
- visual aura.

D. Not better accounted for by another vestibular or ICHD diagnosis.

**Probable vestibular migraine**

A. At least five episodes with vestibular symptoms of moderate or severe intensity, lasting five minutes to 72 hours.

B. Only one of the criteria B and C for vestibular migraine is fulfilled (migraine history *or *migraine features during the episode).

C. Not better accounted for by another vestibular or ICHD diagnosis.

To note: relevant vestibular symptoms are given as spontaneous vertigo, positional vertigo, visually induced vertigo, head motion‐induced vertigo or head motion‐induced dizziness with nausea. Moderate or severe symptoms are those which interfere with, and may prohibit, daily activities. 

### Appendix 2. Neuhauser criteria for migrainous vertigo

**Definite migrainous vertigo**

- Episodic vestibular symptoms of at least moderate severity (rotational vertigo, other illusory self or object motion, positional vertigo, head motion intolerance, i.e., sensation of imbalance or illusory self or object motion that is provoked by head motion) 
- Migraine according to the IHS criteria
- At least one of the following migrainous symptoms during at least two vertiginous attacks: 
  - migrainous headache; 
  - photophobia;
  - phonophobia; 
  - visual or other auras 
- Other causes ruled out by appropriate investigations

**Probable migrainous vertigo**

- Episodic vestibular symptoms of at least moderate severity (rotational vertigo, other illusory self or object motion, positional vertigo, head motion intolerance)
- At least one of the following: 
  - migraine according to the criteria of the IHS; 
  - migrainous symptoms during vertigo; 
  - migraine‐specific precipitants of vertigo, e.g., specific foods, sleep irregularities, hormonal changes; 
  - response to antimigraine drugs
- Other causes ruled out by appropriate investigations

Taken from Neuhauser 2001.

### Appendix 3. Search strategies

The search strategies were designed to identify all relevant studies for a suite of reviews on various interventions for vestibular migraine.

**Table:**

**CENTRAL (CRS)**	**Cochrane ENT Register (CRS)**	**MEDLINE (Ovid)**
1 MeSH DESCRIPTOR Migraine Disorders Explode All AND CENTRAL:TARGET 2 MeSH DESCRIPTOR Vestibular Diseases AND CENTRAL:TARGET 3 MeSH DESCRIPTOR Vertigo AND CENTRAL:TARGET 4 MeSH DESCRIPTOR Dizziness Explode All AND CENTRAL:TARGET 5 #2 OR #3 OR #4 AND CENTRAL:TARGET 6 #1 AND #5 AND CENTRAL:TARGET 7 (migrain* adj5 (vertig* or dizz* or vestibul* or spinning)):AB,EH,KW,KY,MC,MH,TI,TO AND CENTRAL:TARGET 8 #7 OR #6 AND CENTRAL:TARGET	1 MeSH DESCRIPTOR Migraine Disorders Explode All AND INREGISTER 2 MeSH DESCRIPTOR Vestibular Diseases AND INREGISTER 3 MeSH DESCRIPTOR Vertigo AND INREGISTER 4 MeSH DESCRIPTOR Dizziness Explode All AND INREGISTER 5 #2 OR #3 OR #4 AND INREGISTER 6 #1 AND #5 AND INREGISTER 7 (migrain* adj5 (vertig* or dizz* or vestibul* or spinning)):AB,EH,KW,KY,MC,MH,TI,TO AND INREGISTER 8 #7 OR #6 AND INREGISTER 9 * AND CENTRAL:TARGET 10 #8 NOT #9	1 exp Migraine Disorders/ 2 Vestibular Diseases/ 3 Vertigo/ 4 exp Dizziness/ 5 2 or 3 or 4 6 1 and 5 7 (migrain* adj5 (vertig* or dizz* or vestibul* or spinning)).ab,ti. 8 6 or 7 9 randomized controlled trial.pt. 10 controlled clinical trial.pt. 11 randomized.ab. 12 placebo.ab. 13 drug therapy.fs. 14 randomly.ab. 15 trial.ab. 16 groups.ab. 17 9 or 10 or 11 or 12 or 13 or 14 or 15 or 16 18 exp animals/ not humans.sh. 19 17 not 18 20 8 and 19
**Embase (Ovid)**	**Web of Science Core Collection (Web of Knowledge)**	**Trial Registries**
1. exp vestibular migraine/ 2. (migrain* adj5 (vertig* or dizz* or vestibul* or spinning)).ab,ti. 3. 1 or 2 4. Randomized controlled trial/ 5. Controlled clinical study/ 6. Random$.ti,ab. 7. randomization/ 8. intermethod comparison/ 9. placebo.ti,ab. 10. (compare or compared or comparison).ti. 11. ((evaluated or evaluate or evaluating or assessed or assess) and (compare or compared or comparing or comparison)).ab. 12. (open adj label).ti,ab. 13. ((double or single or doubly or singly) adj (blind or blinded or blindly)).ti,ab. 14. double blind procedure/ 15. parallel group$1.ti,ab. 16. (crossover or cross over).ti,ab. 17. ((assign$ or match or matched or allocation) adj5 (alternate or group$1 or intervention$1 or patient$1 or subject$1 or participant$1)).ti,ab. 18. (assigned or allocated).ti,ab. 19. (controlled adj7 (study or design or trial)).ti,ab. 20. (volunteer or volunteers).ti,ab. 21. human experiment/ 22. trial.ti. 23. 4 or 5 or 6 or 7 or 8 or 9 or 10 or 11 or 12 or 13 or 14 or 15 or 16 or 17 or 18 or 19 or 20 or 21 or 22 24. (random$ adj sampl$ adj7 ("cross section$" or questionnaire$1 or survey$ or database$1)).ti,ab. 25. comparative study/ or controlled study/ 26. randomi?ed controlled.ti,ab. 27. randomly assigned.ti,ab. 28. 25 or 26 or 27 29. 24 not 28 30. Cross‐sectional study/ 31. randomized controlled trial/ or controlled clinical study/ or controlled study/ 32. (randomi?ed controlled or control group$1).ti,ab. 33. 31 or 32 34. 30 not 33 35. (((case adj control$) and random$) not randomi?ed controlled).ti,ab. 36. (Systematic review not (trial or study)).ti. 37. (nonrandom$ not random$).ti,ab. 38. "Random field$".ti,ab. 39. (random cluster adj3 sampl$).ti,ab. 40. (review.ab. and review.pt.) not trial.ti. 41. "we searched".ab. 42. review.ti. or review.pt. 43. 41 and 42 44. "update review".ab. 45. (databases adj4 searched).ab. 46. (rat or rats or mouse or mice or swine or porcine or murine or sheep or lambs or pigs or piglets or rabbit or rabbits or cat or cats or dog or dogs or cattle or bovine or monkey or monkeys or trout or marmoset$1).ti. and animal experiment/ 47. 29 or 34 or 35 or 36 or 37 or 38 or 39 or 40 or 43 or 44 or 45 48. 23 not 47 49. 3 and 48	# 3 #2 AND #1 Indexes=SCI‐EXPANDED, CPCI‐S Timespan=All years # 2 TOPIC: (((randomised OR randomized OR randomisation OR randomisation OR placebo* OR (random* AND (allocat* OR assign*) ) OR (blind* AND (single OR double OR treble OR triple) )))) Indexes=SCI‐EXPANDED, CPCI‐S Timespan=All years # 1 TOPIC: (migrain* NEAR/5 (vertig* or dizz* or vestibul* or spinning) ) Indexes=SCI‐EXPANDED, CPCI‐S Timespan=All years	**Clinicaltrials.gov** ( migraine OR migrainous ) AND ( vertigo OR dizziness OR dizzy OR vertiginous OR vestibular OR spinning ) **ICTRP** migrain* AND (vertig* OR dizz* OR vestibul* OR spinning)

### Appendix 4. Trustworthiness Screening Tool

This screening tool has been developed by Cochrane Pregnancy and Childbirth. It includes a set of predefined criteria to select studies that, based on available information, are deemed to be sufficiently trustworthy to be included in the analysis. These criteria are:

**Research governance**

- Are there any retraction notices or expressions of concern listed on the Retraction Watch Database relating to this study?
- Was the study prospectively registered (for those studies published after 2010)? If not, was there a plausible reason?
- When requested, did the trial authors provide/share the protocol and/or ethics approval letter?
- Did the trial authors engage in communication with the Cochrane Review authors within the agreed timelines?
- Did the trial authors provide IPD data upon request? If not, was there a plausible reason?

**Baseline characteristics**

- Is the study free from characteristics of the study participants that appear too similar (e.g. distribution of the mean (SD) excessively narrow or excessively wide, as noted by Carlisle 2017)?

**Feasibility**

- Is the study free from characteristics that could be implausible? (e.g. large numbers of women with a rare condition (such as severe cholestasis in pregnancy) recruited within 12 months);
- In cases with (close to) zero losses to follow‐up, is there a plausible explanation?

**Results**

- Is the study free from results that could be implausible? (e.g. massive risk reduction for main outcomes with small sample size)?
- Do the numbers randomised to each group suggest that adequate randomisation methods were used (e.g. is the study free from issues such as unexpectedly even numbers of women ‘randomised’ including a mismatch between the numbers and the methods, if the authors say ‘no blocking was used’ but still end up with equal numbers, or if the authors say they used ‘blocks of 4’ but the final numbers differ by 6)?

Studies assessed as being potentially ‘high risk’ will be not be included in the review. Where a study is classified as ‘high risk’ for one or more of the above criteria we will attempt to contact the study authors to address any possible lack of information/concerns. If adequate information remains unavailable, the study will remain in ‘awaiting classification’ and the reasons and communications with the author (or lack of) described in detail.

The process is described in full in Figure 2. 

## Data and analyses

**Comparison 1:** Triptans versus placebo

Outcome or subgroup title	No. of studies	No. of participants	Statistical method	Effect size
1.1 Improvement in vertigo (global score)	2		Risk Ratio (M‐H, Random, 95% CI)	Subtotals only
1.1.1 Up to 2 hours	2	262	Risk Ratio (M‐H, Random, 95% CI)	0.84 [0.66, 1.07]
1.1.2 > 12 hours	1	170	Risk Ratio (M‐H, Random, 95% CI)	1.01 [0.90, 1.12]
1.2 Improvement in headache	2		Risk Ratio (M‐H, Random, 95% CI)	Subtotals only
1.2.1 Up to 2 hours	2	248	Risk Ratio (M‐H, Random, 95% CI)	0.69 [0.49, 0.96]
1.2.2 > 12 hours	1	171	Risk Ratio (M‐H, Random, 95% CI)	1.16 [0.99, 1.37]
1.3 Improvement in other migrainous symptoms	1		Risk Ratio (M‐H, Random, 95% CI)	Subtotals only
1.3.1 Nausea and vomiting at up to 2 hours	1	239	Risk Ratio (M‐H, Random, 95% CI)	0.80 [0.62, 1.03]
1.3.2 Nausea and vomiting at > 12 to 72 hours	1	170	Risk Ratio (M‐H, Random, 95% CI)	1.02 [0.93, 1.11]
1.3.3 Photo‐ and phonophobia at up to 2 hours	1	240	Risk Ratio (M‐H, Random, 95% CI)	1.05 [0.75, 1.47]
1.3.4 Photo‐ and phonophobia at > 12 to 72 hours	1	171	Risk Ratio (M‐H, Random, 95% CI)	1.20 [1.01, 1.42]
1.4 Other adverse effects	1		Risk Ratio (M‐H, Random, 95% CI)	Subtotals only
1.4.1 Gastrointestinal disturbance	1	114	Risk Ratio (M‐H, Random, 95% CI)	1.24 [0.60, 2.56]
1.4.2 Sleep disturbance	1	114	Risk Ratio (M‐H, Random, 95% CI)	2.41 [1.43, 4.07]
1.4.3 Cardiovascular side effects	1	114	Risk Ratio (M‐H, Random, 95% CI)	0.87 [0.22, 3.44]
1.4.4 Paraesthesia, flushing, warm or hot sensations	1	114	Risk Ratio (M‐H, Random, 95% CI)	0.73 [0.25, 2.14]
1.4.5 Headache	1	114	Risk Ratio (M‐H, Random, 95% CI)	0.75 [0.35, 1.60]

## Characteristics of studies

### Characteristics of included studies [ordered by study ID]

**Table:** NCT02447991

***Study characteristics***		
Methods	Double‐blind, placebo‐controlled, parallel‐group RCT Duration of treatment: maximum of 3 episodes of vestibular migraine Duration of follow‐up: until 3 attacks have been treated; up to 4 years, as required	
Participants	**Setting:** 2‐centre trial based in the USA at tertiary neurology centres **Sample size:** **Number randomised:** 114 participants reported in the results (discrepancy here with trial registry site, which states that 134 participants were randomised) **Number completed:** 94 participants **Participant baseline characteristics** **Age: ** Described as between 18 and 65 years, but no further details are provided **Gender: ** Rizatriptan group: 69 female (77.5%): 20 male (22.5%) Placebo group: 32 female (71.1%): 13 male (28.9%) **Probable/definite vestibular migraine: ** Inclusion criterion is definite vestibular migraine **Attack frequency at baseline: ** At least 2 migraine attacks in the preceding 12 months; no other information provided **Duration of disease:** Not reported **Inclusion criteria:** Age ≥ 18 years and ≤ 65 years. A history that fulfils all criteria for vestibular migraine. Episodes must have a spontaneous onset and resolution without associated hearing loss or interictal neurotologic deficits. Other causes of vestibular symptoms ruled out by appropriate clinical investigations. Current medication list compatible with concomitant medications. Able to maintain a vestibular symptom diary and complete all other study procedures. At least 2 vestibular migraine attacks during the 12‐month observation period. **Exclusion criteria:** Ménière’s disease. Migraine with brainstem aura (formerly basilar‐type migraine) by ICHD‐3 criteria. Ischaemic heart disease, coronary artery vasospasm, uncontrolled hypertension. History of stroke or transient ischaemic attack. History of using rizatriptan specifically to treat vestibular attacks. History of adverse response to triptans. Women who are pregnant or breastfeeding. Unable or unwilling to comply with study requirements for any reason. **Diagnosis of vestibular migraine:** International Headache Society criteria for definite vestibular migraine (see Appendix 1)	
Interventions	**Intervention (n = 89 randomised, n = 59 completed)** Rizatriptan 10 mg capsule to be taken orally during 1 acute episode, until 3 episodes had been treated with the study drug **Comparator (n = 45 randomised, n = 35 completed)** Placebo capsules (contents not stated) to be taken orally during 1 acute episode, until 3 episodes had been treated with the study drug **Background interventions administered to all participants** None reported Additional treatments (for migraine prophylaxis or other conditions) were permitted if they had been taken during the run‐in period to the trial and participants were on a stable dose	
Outcomes	**Primary outcomes relevant to this review:** **Improvement in vertigo** Participants reported symptoms using a patient self‐report of the severity of vestibular symptoms (vertigo and unsteadiness/dizziness) where: 0 = no symptoms 1 = mild symptoms (no interference with activities) 2 = moderate symptoms (had to alter some activities) 3 = severe symptoms (had to stop most or all activities) Improvement was considered a reduction in symptoms from moderate/severe to none/mild **Change in vertigo** Not reported **Serious adverse events** Assessed and reported. Defined as those events that result in death, are life‐threatening, require inpatient hospitalisation or prolongation of existing hospitalisation, create persistent or significant disability/incapacity, or cause a congenital anomaly or birth defects. **Secondary outcomes relevant to this review:** **Disease‐specific health‐related quality of life** Not reported **Improvement in headache** Assessed using the same scale as that used for vertigo (0 to 3, where 3 = most severe symptoms) Improvement was considered a reduction in symptoms from moderate/severe to none/mild **Improvement in other migrainous symptoms ** Assessed using the same scale as that used for vertigo (0 to 3, where 3 = most severe symptoms). Symptoms assessed included nausea/vomiting and photo/phonophobia. Improvement was considered a reduction in symptoms from moderate/severe to none/mild **Other adverse effects** Assessed and reported **Other outcomes reported in the study:** Sensitivity to motion Satisfaction with treatment Health‐related quality of life by Short Form Survey ‐ 12 (SF‐12)	
Notes	**Research integrity checklist:** No retractions or expressions of concern were identified Baseline characteristics of the groups are not fully reported; no concerns based on the (limited) data available Loss to follow‐up is not fully described, but some losses are noted No implausible results were identified No concerns over randomisation	
***Risk of bias***		
**Bias**	**Authors' judgement**	**Support for judgement**
Random sequence generation (selection bias)	Unclear risk	Quote: "A randomization scheme will be developed to assign the 120 patients expected to be included in the Treatment Phase to parallel treatment arms in a 2:1 ratio of active drug:placebo." Comment: no information is provided regarding generation of the randomisation sequence.
Allocation concealment (selection bias)	Unclear risk	Comment: no information regarding how the randomisation process would occur and whether the randomisation list would be adequately concealed.
Blinding of participants and personnel (performance bias) All outcomes	Low risk	Comment: trial is reported to be blinded to participants and study personnel.
Blinding of outcome assessment (detection bias) All outcomes	Low risk	Comment: self‐reported symptoms by blinded participants.
Incomplete outcome data (attrition bias) All outcomes	High risk	Comment: dropout was 22.2% in the placebo arm and 33.7% in the intervention arm, although some participants did provide partial follow‐up data. This is sufficient to impact the results of the trial, and may introduce the potential for bias in the results.
Selective reporting (reporting bias)	Low risk	Comment: outcomes are fully reported on the trial registry website according to the pre‐specified plan.
Other bias	Low risk	Comment: no other concerns identified.

**Table:** Neuhauser 2003

***Study characteristics***		
Methods	Randomised, double‐blind, placebo‐controlled, cross‐over (after one attack) trial Total duration of treatment and follow‐up not reported	
Participants	**Setting: ** Specialist dizziness neurology clinic, hospital outpatient management in Germany (single‐centre). Study dates not reported. **Sample size:** **Number randomised: **19 participants **Number completed: **16 participants (although only 10 participants experienced attacks) **Participant baseline characteristics** Only reported for those who completed the trial **Age: ** Zolmitriptan group: range 29 to 57 years Placebo group: range 29 to 57 years **Gender: ** Zolmitriptan group: 7 females: 1 male Placebo group: 7 females: 2 males **Probable/definite vestibular migraine: ** Not reported (study was published before the IHS criteria were defined) **Attack frequency at baseline: ** At least 2 attacks in the preceding 12 months; no other information provided **Duration of disease:** Not reported **Inclusion criteria:** A diagnosis of vestibular migraine. At least 2 episodes of migrainous vertigo lasting longer than 2 hours within 12 months before enrollment. Aged between 18 and 65 years. **Exclusion criteria:** Vestibular disorders other than vestibular migraine. Non‐vertiginous dizziness (e.g. orthostatic or due to panic disorder) unless it could be clearly differentiated from attacks of VM by the patient. History of basilar, ophthalmoplegic or hemiplegic migraine. Severe impairment of hepatic or renal function. Symptoms suggestive of ischaemic heart disease. History of stroke or transitory ischaemic attacks. Alcohol or drug abuse. Diastolic blood pressure more than 95 mmHg or systolic more than 160 mmHg. No contraception for women of childbearing age. Hypersensitivity to the study medication. In addition, patients should not use a triptan or ergot derivative within 24 hours before and after taking study medication; change or start any prophylactic migraine treatment 3 months before taking study medication; and take any other anti‐vertiginous drugs or pain medication within 6 hours before taking study medication. **Diagnosis of vestibular migraine:** Criteria were: 1) episodic vestibular symptoms of at least moderate severity (rotational vertigo, other illusory self‐ or object‐motion, positional vertigo or head motion intolerance, i.e. sensation of imbalance or illusory motion provoked by head movements). Vestibular symptoms were “moderate” if they interfered with but did not prohibit daily activities, “severe” if patients could not continue daily activities; 2) current or previous history of migraine according to the 1988 criteria of the International Headache Society; 3) one of the following migrainous symptoms during at least 2 vertiginous attacks: migrainous headache, photophobia, phonophobia, visual or other auras; and 4) other causes ruled out by appropriate investigations.	
Interventions	**Intervention (n = 11 randomised, number completed not reported)** Zolmitriptan 2.5 mg to be taken orally when symptoms of vestibular migraine were moderate or severe **Comparator (n = 8 randomised, number completed not reported)** A placebo of identical appearance and taste was to be taken when symptoms of vestibular migraine were moderate or severe **Background interventions administered to all participants** A second dose of study medication (either triptan or placebo, accordingly) or a rescue medication (dimenhydrinate 150 mg for vertigo and paracetamol 500 mg for headache) could be taken after 2 hours	
Outcomes	**Primary outcomes relevant to this review:** **Improvement in vertigo** Vertigo was measured as mild, moderate, severe or no vertigo. Improvement was counted as vertigo severity changing from moderate or severe to mild or no vertigo. Patients reported symptoms in a structured diary. **Change in vertigo** Not reported **Serious adverse events** Not reported **Secondary outcomes relevant to this review:** **Disease‐specific health‐related quality of life** Not reported **Improvement in headache** Assessed using the same score as vertigo. Improvement was counted as headache changing from moderate or severe to mild or no headache. Patients reported symptoms in a structured diary. **Improvement in other migrainous symptoms ** Not reported **Other adverse effects** Not reported **Other outcomes reported in the study:** No further outcomes were reported	
Notes	**Research integrity checklist:** No retractions/expressions of concern were identified Trial registration was not required, as this article was published before 2010 Plausible loss to follow‐up was reported No implausible results were noted The numbers allocated to each group do not suggest any issues with randomisation	
***Risk of bias***		
**Bias**	**Authors' judgement**	**Support for judgement**
Random sequence generation (selection bias)	Unclear risk	Comment: no information given on randomisation sequence generation.
Allocation concealment (selection bias)	Unclear risk	Comment: no information given on allocation concealment.
Blinding of participants and personnel (performance bias) All outcomes	Unclear risk	Comment: stated to be "double‐blind" and placebo was used. No other information given on blinding of study personnel.
Blinding of outcome assessment (detection bias) All outcomes	Low risk	Quote: "placebo of identical appearance and taste" Comment: primary outcome measures were self‐reported symptoms by blinded participants.
Incomplete outcome data (attrition bias) All outcomes	High risk	Comment: 3 withdrawals from the study (only 19 participants in total). In addition, 6 participants who did not suffer an attack during the study period (which is undefined) were also excluded from analysis, therefore further reduction in sample size.
Selective reporting (reporting bias)	High risk	Comment: data are not reported in a way that accounts for the cross‐over design of this trial, therefore it is impossible to appropriately assess correlation in outcomes for an individual participant.
Other bias	Low risk	Comment: no other concerns identified.

ICHD: International Classification of Headache Disorders; IHS: International Headache Society; RCT: randomised controlled trial; VM: vestibular migraine

### Characteristics of excluded studies [ordered by study ID]

**Table:**

Study	Reason for exclusion
ACTRN12616000683437	This study is not an RCT.
Byun 2021	This article is a systematic review, not an RCT. The reference list has been checked to ensure that any relevant RCTs are included in this review.
ChiCTR1800014766	This study is not an RCT.
Furman 2009	This study is not an assessment of medications used to treat acute attacks of vestibular migraine. Instead, it aims to assess whether prophylactic treatment with triptans might improve motion sickness symptoms in those with vestibular migraine, when subjected to a vestibular stimulus.
Furman 2011	This study is not an assessment of medications used to treat acute attacks of vestibular migraine. Instead, it aimed to assess whether prophylactic treatment with triptans might improve motion sickness symptoms in those with vestibular migraine, when subjected to a vestibular stimulus.
Hou 2020	This is a systematic review, not an RCT. The reference list has been checked to ensure that any relevant articles have been included in this review.
Marcus 2006	This study is not an assessment of medications used to treat acute attacks of vestibular migraine. Instead, it aimed to assess whether prophylactic treatment with triptans might improve motion sickness symptoms in those with vestibular migraine, when subjected to a vestibular stimulus.

RCT: randomised controlled trial

## Differences between protocol and review

We noted a discrepancy in the protocol with regard to the inclusion of cross‐over trials (Webster 2022c). Under Types of studies we had stated that cross‐over trials would be included. However, under Unit of analysis issues we had erroneously stated that only data from the first phase of the study would be included. As acute attacks of vestibular migraine are discrete events, we considered that cross‐over trials would be appropriate for inclusion in this review, providing we could account for the correlation in the data. We did identify one cross‐over trial (Neuhauser 2003). Although we were unable to account for the correlation in the data we did include the data from this study, as we considered that any estimate of the effect would be conservative (see Unit of analysis issues for more information). 

For the outcome 'improvement of other migrainous symptoms' we planned to pool different symptoms (such as nausea and vomiting, photophobia and phonophobia, visual aura) and assess this as a composite measure. However, we also planned to explore whether the different symptoms may be affected differently by the intervention, using subgroup analysis. As a single study assessed these outcomes, we considered it more straightforward to present these outcomes individually, rather than attempt to convert them to a pooled outcome. 
